# Case report: *Providencia stuartii* conjunctivitis

**DOI:** 10.1186/s12348-016-0097-9

**Published:** 2016-08-16

**Authors:** Elliot S. Crane, May Shum, David S. Chu

**Affiliations:** 1 Institute of Ophthalmology and Visual Science, New Jersey Medical School, Rutgers University, Doctors Office Center, Suite 6100, 90 Bergen Street, Newark, NJ 07103 USA; 2 Metropolitan Eye Research and Surgery Institute, 540 Bergen Blvd, Suite D, Palisades Park, NJ 07650 USA

**Keywords:** Bacterial, Conjunctivitis, Rare, *Providencia stuartii*

## Abstract

**Purpose:**

The purpose of this study is to report a case of *Providencia stuartii* conjunctivitis.

**Methods:**

This study is a retrospective chart review of a patient with persistent conjunctivitis.

**Results:**

We report the first case of *P. stuartii* conjunctivitis. Our patient was an elderly man living in a nursing home who was likely immunocompromised from longstanding diabetes mellitus. A conjunctival swab culture was able to identify the infecting bacteria and its antibiotic susceptibility. The conjunctivitis was successfully treated with vancomycin drops and oral sulfamethoxazole and trimethoprim.

**Discussion:**

*P. stuartii* is an increasingly common bacterium found in the urine of immunocompromised nursing home residents with indwelling Foley catheters. While it has rarely been found to cause ocular infections, *P. stuartii* may be suspected in elderly, immunocompromised nursing home residents.

## Introduction

The genus Providencia includes five facultative gram-negative bacilli, of which *Providencia stuartii* is the species most commonly causing infection [1]. *P. stuartii* has been increasingly isolated from urine cultures in nursing home residents with long-term urinary catheters [2]. It is particularly common in patients with blocked indwelling urinary catheters [3] and is also an uncommon cause of bacteremia [1].

Although we could not find any published cases of *P. stuartii* conjunctivitis, we did find two mentions of *P. stuartii* keratitis; in both cases, they were listed as one line in a chart of etiologic agents without further comment [4, 5]. Koreishi et al. also detailed five ocular infections by a closely related bacteria, *Providencia rettgeri* [6].

We report a case of *P. stuartii* conjunctivitis in an elderly man living in a nursing home in New Jersey.

## Case report

A 74-year-old Asian man with diabetes mellitus and glaucoma presented with a 10-month history of worsening vision in his right eye with persistent ocular discharge, pruritus, and irritation. He had an 18-year history of poorly controlled type II diabetes mellitus complicated by end-stage renal disease and diabetic retinopathy. His ophthalmic history included bilateral proliferative diabetic retinopathy (treated with pan-retinal photocoagulation) and bilateral neovascular and chronic angle-closure glaucoma (treated with pressure reduction surgery in his right eye and timolol drops bilaterally). His ophthalmic surgical history included bilateral cataract extraction with posterior chamber intraocular lens (PC IOL) implantation 10 years prior to presentation and two Baerveldt tube insertions in his right eye that were both removed due to hypotony and exposure over 2 years prior to presentation. He has no history of contact lens wear but was a former smoker and is a nursing home resident; he has taken a moxifloxacin hydrochloride table daily since his glaucoma drainage device surgeries. Two years prior to presentation, his visual acuity (VA) was 20/200 in the right eye (RE) and no light perception (NLP) in the left eye (LE).

**Fig. 1:**
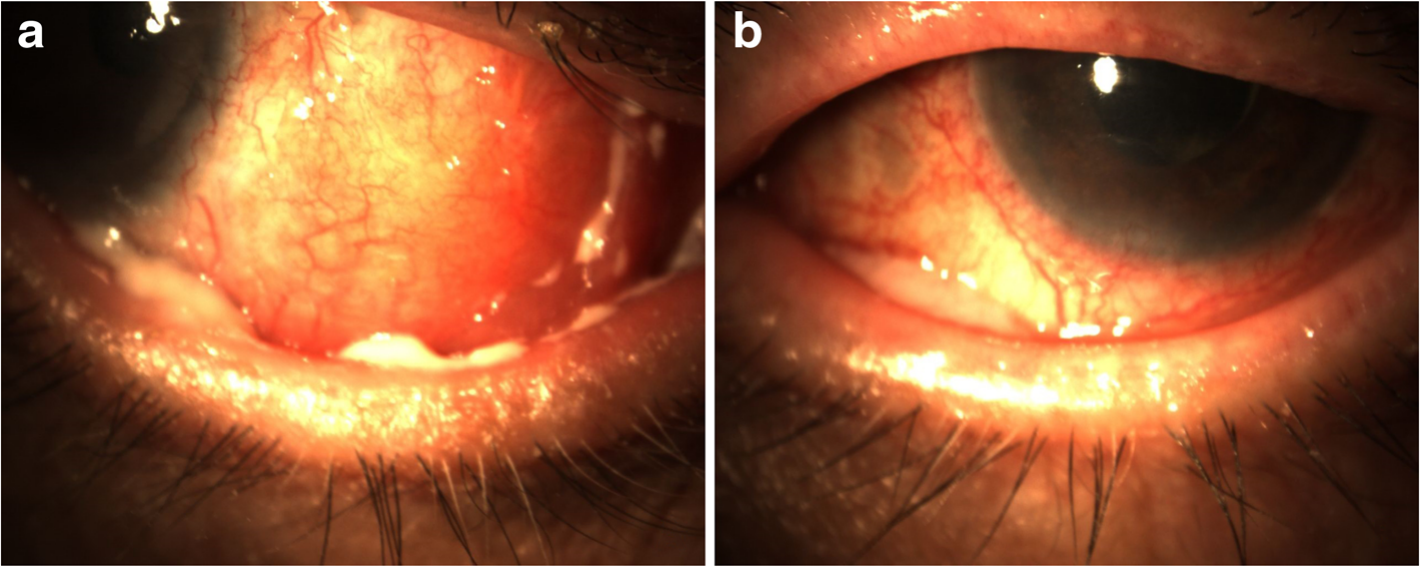
**a** RE and **b** LE anterior segment photographs showing injected and edematous conjunctiva with mucoid discharge

At presentation, his VA was hand motion in the RE and NLP in the LE. His RE was found to have mucopurulent conjunctivitis (Fig. 1a). The conjunctiva in his LE was clear and quiet. Cultures were taken, and his RE was treated with a betadine wash (in office), vancomycin drops every 2 h, and fortified tobramycin drops every 2 h.

Three weeks later, the patient reported that his RE subjectively improved. On exam, the conjunctiva in his RE was less inflamed but the VA worsened to light perception (LP). Additionally, the conjunctivitis was found to have spread to his LE (Fig. 1b). RE cultures grew *P. stuartii* susceptible to: piperacillin/tazobactam, ceftazidime, ceftriaxone, cefepime, ertapenem, and trimethoprim/sulfamethoxazole. Cultures were also taken from the LE, which later showed no growth. His vancomycin drops were extended bilaterally, and oral sulfamethoxazole and trimethoprim were started with dose adjusted to his renal clearance. His fortified tobramycin drops were discontinued due to *P. stuartii* resistance.

**Fig. 2:**
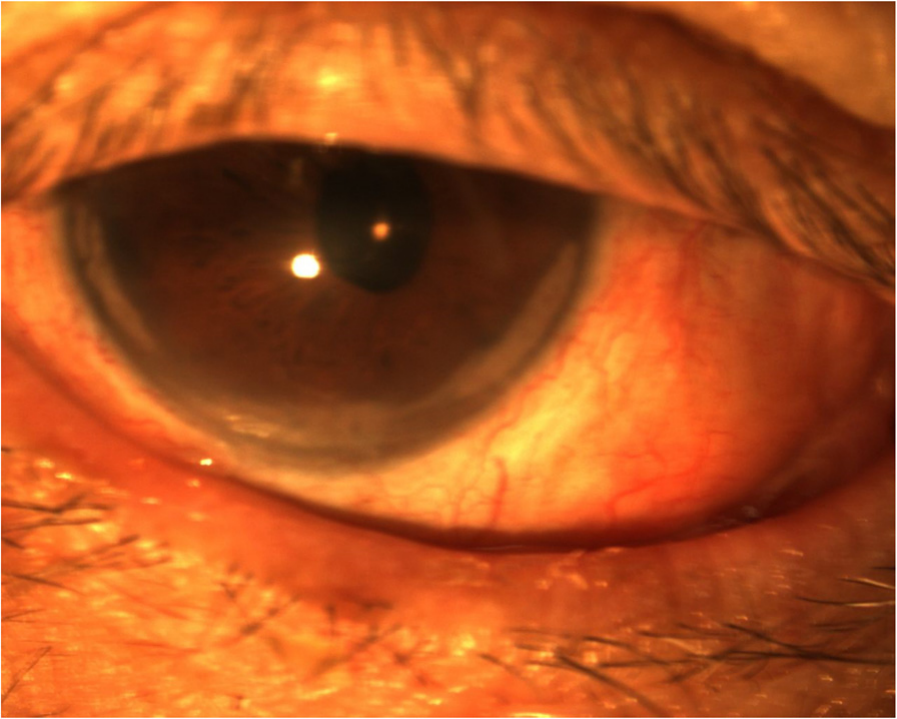
Anterior segment photograph showing minimal residual conjunctival injection

Two months after initial presentation, his conjunctivitis had almost completely resolved bilaterally (Fig. 2). His VA remained NLP in his LE, but his RE VA improved to count fingers.

## Discussion

*P. stuartii* is an increasingly common bacterium found in the urine of nursing home residents with indwelling Foley catheters [2]. In a 12-year study from a large community hospital in Ohio, *P. stuartii* was found to cause bacteremia in 49 patients [1]. Of these, 78 % were over 70 years of age, 96 % were admitted to the hospital from a nursing home, and 92 % had a long-term indwelling Foley catheter when admitted to the hospital. Our patient was over 70 years old and lived in a nursing home but did not use an indwelling Foley catheter as he had end-stage renal disease.

We could only find two cases of ocular infection due to *P. stuartii* in the published literature. Both instances were cases of keratitis rather than conjunctivitis, and neither provided detailed information on the individual *P. stuartii* infection. Sun et al. described *P. stuartii* keratitis in the right eye of a 13-year-old girl who had worn an orthokeratology lens for 2 years and presented with a corneal ulcer [4]. The textbook, *Smolin and Thoft’s The Cornea: Scientific Foundations and Clinical Practice*, provides an original chart of gram-negative bacterial isolates from keratitis from Pittsburgh, PA [5]. They found one case of keratitis from *P. stuartii* that was not susceptible to any of the tested antibiotics.

Koreishi et al. described five ocular infections from a related bacterium, *Providencia rettgeri* [6]. They detailed two cases of keratitis, one of dacryocystitis, one of conjunctivitis, and one of conjunctivitis/endophthalmitis. All five of their subjects were elderly. Three of their subjects were either locally or systemically immunocompromised (one of their subjects had diabetes and used topical steroids, a second used topical steroids, and a third was being treated with methotrexate). Two of their patients had positive urinary analyses (leukocytosis in one and bacteria in the other), but neither was cultured. They found that concurrent urinary tract infections and an immunocompromised state were likely risk factors.

Similar to other patients infected with *P. Stuartii*, our patient was an elderly man living in a nursing home who was likely immunocompromised from diabetes mellitus. We could not confirm if his urinary tract was infected with *P. stuartii* as he produced no urine that could be cultured. Our patient’s conjunctivitis was successfully treated with vancomycin drops, oral sulfamethoxazole and trimethoprim, and oral moxifloxacin hydrochloride. We found that conjunctival swab cultures were instrumental in identifying the infecting bacteria and its antibiotic susceptibility.

In summary, *P. stuartii* is an uncommon cause of ocular infections, but may be suspected in elderly, immunocompromised nursing home residents.

## Abbreviations

LE, left eye; LP, light perception; NLP, no light perception; PC IOL, posterior chamber intraocular lens; RE, right eye; VA, visual acuity
